# Predicting the Distribution and Dry Matter Intake of Cow Pens with Machine Learning

**DOI:** 10.3390/ani16203184

**Published:** 2026-10-10

**Authors:** Padraig M. Lucey, Ian J. Lean, Nadiia Stepanchenko, Leoni F. Martins, Alexander N. Hristov, Heidi A. Rossow

**Affiliations:** 1 Veterinary Medicine Teaching and Research Center, School of Veterinary Medicine, University of California, Davis, Tulare, CA 93274, USA; 2 Scibus, Camden, NSW 2570, Australia; 3 Department of Animal Science, The Pennsylvania State University, University Park, PA 16802, USA

**Keywords:** dry matter intake prediction, distribution shape, precision ration formulation

## Abstract

Current rations are formulated on the requirements of a representative cow plus a correction factor. This is not precision feeding because in practice cows are fed as a pen, and within a pen, dry matter intake (DMI) among cows is not uniform and has skew or kurtosis. Since ration formulation depends on DMI as a binding constraint, rations formulated to the pen DMI could be more precise if the distribution of DMI is better described. The objectives of this study were to: 1. Develop an algorithm, the distribution shape model (DSM), to predict pen DMI based on the distribution of cow measures which could easily be estimated on a commercial dairy farm; and 2. Compare predictions of pen DMI from the DSM to predictions of pen DMI based on a representative cow using the National Academies of Sciences, Engineering, and Medicine (NASEM) equation model (NSM). Individual cow DMI from previously published studies was used to train an Extreme Gradient Boosting (XGB) algorithm to predict the distribution shape of pen DMI (Beta or Generalized Normal) from parity, week in milk, and descriptive statistics of milk yield. The NSM predicted DMI for a representative cow from pen-average inputs, and this prediction was multiplied by the number of cows, simulating the scenario where DMI is assumed to be uniform across a pen. Both models were tested by comparing predicted with observed total pen DMI using an independent testing dataset of 229,909 pens generated from previously collected cow observations. For pens classified as Beta, the mean squared error of prediction (MSEP) of pen DMI from the DSM and NSM was 5249 and 9736 kg^2^, respectively (root mean squared error of prediction, RMSEP, 72.5 and 98.7 kg/d), with 3.0% and 80.2% due to mean bias, 5.4% and 8.3% due to regression slope not equal to 1, and 91.6% and 11.5% due to random variation. For pens classified as Generalized Normal, MSEP was 2086 and 6431 kg^2^ (RMSEP 45.7 and 80.2 kg/d), with 0.8% and 73.2% due to mean bias, 7.6% and 4.7% due to slope not equal to 1, and 91.6% and 22.1% due to random variation. The NSM consistently overpredicted pen DMI, contributing to its higher error. Including the distribution shape in the prediction of DMI improved precision and accuracy of pen DMI prediction and should be considered when estimating the total daily DMI fed to a pen.

## Simple Summary

Improving diet formulation accuracy and precision is essential to profitability and sustainability of dairy farms. Diets are currently formulated for a reference cow which ignores variation in nutrient requirements among cows and over time. This work developed a machine learning model that predicts the distribution of dry matter intake in pens. Using individual cow milk yield, week in milk and parity, the machine learning model improved dry matter intake prediction compared to an empirical equation. By accounting for the distribution of dry matter intake, we accurately and precisely predicted pen dry matter intake which could improve economic efficiency and sustainability by reducing feed waste.

## 1. Introduction

Individual cow dry matter intake (DMI) is difficult to measure on a commercial dairy with large pens. The number of cows in the pen is not always known and depends on the day relative to when cows are moved in and out of pens on the dairy. Individual feed bins are impractical and cost-prohibitive; spatial imaging attempts to predict individual DMI are yet to be validated or implemented [1]. Common practice is to use the pen mean DMI as input values in diet formulation [2], but most pens contain cows with a broad range of days in milk, milk yield, and nutrient requirements within the pen which are not normally distributed. In [3], the Beta distribution was reported as the most common shape of DMI for dairy pens of multiple types. This shows the mean is limited as an input value as it does not explain distribution shape, skew or kurtosis. A robust method of estimating DMI distribution shape without knowledge of individual cow DMI will improve diet precision and accuracy.

Cow DMI has been calculated by formulas using biological and environmental information such as milk yield, milk energy, mud depth, body condition score and bodyweight [4,5]. This is constrained by the relevant input information being available from commercial dairies [6]. Lactating cows are usually grouped in pens by week in milk (WIM), parity, reproductive status, or milk production [7], which affects the distribution of cows’ nutrient requirements. There are biologically plausible relationships between cow factors, such as milk yield and DMI, facilitating the empirical deduction of DMI equations. But the minimum or maximum WIM, milk yield, or parity of a pen does not have a biological relationship to the distribution that fits the DMI. Since empirical modeling assumes biological plausibility, the possibility of constructing a precise pen level model empirically is limited. Instead, techniques such as machine learning algorithms could connect these arbitrary relationships [8].

The objectives of this study were to construct and compare two models of pen DMI prediction. First, a large training dataset of pens based on real cow weekly observations was created. Then, a distribution shape model (DSM) was developed by training machine learning algorithms to predict the distribution shape of DMI in a pen using these training data. Thirdly, the National Academies of Sciences, Engineering, and Medicine (NASEM) equation model (NSM) was used to predict the DMI of each pen. Both the DSM and NSM model predictions of pen DMI were compared to the observed pen DMI using a testing dataset constructed from one independent external dataset. This modeling study will test if the pen DMI predicted by the DSM will be closer to the true value than the NSM by having lower predictive model error.

## 2. Materials and Methods

To be included in the training or testing datasets, each individual cow observation required an identifier, parity, WIM, average weekly DMI and average weekly milk yield. Some datasets also included milk protein, milk fat, body condition score (BCS) or bodyweight (BW). All variables were tested for outliers and records were removed if variables milk fat, milk protein were greater than the 99th percentile. For the training dataset, 9 studies including 9029 weekly observations of 630 cows (Table 1) with a maximum WIM of 45 weeks were included. The Lean dataset [9] was excluded from model development and reserved as an independent testing dataset.

After the exclusion of outliers, or observations with missing DMI or milk yield from the training dataset, individual weekly cow observations were randomly sorted into different pen sizes ranging from 25 to 250 cows to create 4 pen types typical of commercial dairies labeled Fresh, Early, Peak and Late. These pens were generated from real cow observations by sampling observations within different WIM intervals with different combinations of starting and ending weeks (Table 2). For example, Fresh pens were generated with WIM intervals of 1–3, 1–4, and 1–5 weeks, while Early pens began at week 1 and had a wide upper limit to cover the full lactation. Pen generation was performed using Python 3.10 (Python Software Foundation, Fredericksburg, VA, USA, https://www.python.org/). Duplicate cow records were not allowed within any pen. Cow observations were reused across different pens in both the training and testing datasets. A total of 7,191,040 training pens were generated (Table 2). After removal of outliers, cow observations from the testing dataset (Lean [9]) were used to similarly generate 280,740 testing pens to evaluate the total DMI predictions of both DSM and NSM.

Feature selection focused on variables that could be easily available on a commercial dairy farm. The features considered for training the machine learning algorithms included pen size and parity makeup, with within-pen descriptive statistics of milk yield, milk fat, milk protein, BW, BCS and WIM. These included the mean, median, minimum, maximum, 5th, 25th, 75th and 95th percentiles, standard deviation, skew and kurtosis (skew and kurtosis omitted for WIM). Missing individual cow values of these variables were excluded from the calculations, except for milk yield, parity, and WIM as these had no missing data. Percentiles summarized the distribution of each variable, the mean and median described central tendency, and skew and kurtosis described asymmetry and tail behavior, respectively.

To develop the DSM, prediction of pen DMI was performed in 2 steps. First, an Extreme Gradient Boosting (XGB) classification model predicted the pen DMI distribution family (Beta or Generalized Normal). Second, separate XGB regression models predicted the parameter values for the Beta or Generalized Normal distribution. Preliminary analysis of these data demonstrated the Beta and Generalized Normal were the most frequent shapes to describe pen DMI from a candidate pool of 60 shapes (Supplementary Table S1), so fitting was restricted to these distributions. Candidate distributions were compared by how often they produced the highest Kolmogorov–Smirnov test *p*-value [19]. To label the testing and training datasets, both distributions were fit to the observed cow DMI of each pen using maximum likelihood estimation. Each pen was assigned to one of the two based on the larger unrounded Kolmogorov–Smirnov goodness-of-fit test *p*-value (*p* > 0.05) with the SciPy package [20] in Python 3.10; no separate tie-breaking rule was applied. If fitting failed or neither distribution achieved *p* > 0.05, that pen generation run was skipped.

The Beta distribution had four parameters; two shape (α and β), location and scale, which explain symmetry, peak location, spread, skewness and dispersion of data. The three parameters of the Generalized Normal were shape (tails), location, and scale. These parameters defined the probability density function (PDF) of DMI for each pen. Beta distributions can represent skewed or symmetrical data. The Generalized Normal is the normal distribution with an additional shape parameter and can describe data with a concentration around the mean, and different tails than the normal distribution.

Training pens were filtered for extreme milk descriptors (kurtosis < 15), removing 3644 pens. After visual assessment of fitted distributions, parameter value limits were applied to exclude implausible distribution curves. Beta fits were retained when shape a < 80, shape b < 50 and location > −60 in training pens, and shape a < 20, shape b < 25 and location > −30 in testing pens. Generalized Normal fits were retained when shape < 80 and location < 50 in training pens, and shape < 20 and location < 25 in testing pens. Parameter filtering removed 1,983,711 training pens and 50,831 testing pens, leaving 5,203,685 and 229,909 pens, respectively. Of these training pens, 871,956 (16.8%) contained no observed cow BCS. These missing BCS descriptors were imputed with the mean of that descriptor across all other pens. This allowed pens without BCS observations to be included when evaluating BCS features.

The labeled training dataset of pens was randomly split 75:25 using the “train_test_split” function of Scikit-learn [21]. Cow observations were reused across pens, so the two subsets shared source observations. The XGB classifier was trained using the pen features and labels of the 75% subset. Classification model performance was assessed on the remaining 25% of the training data (training–testing) by comparing the predicted label to the assigned distribution label for accuracy, precision, recall and F1-score. Separate XGB regression models were trained to predict the parameter values of the Beta and Generalized Normal distribution shapes from the pen features. These target values were obtained from the maximum-likelihood fitting of the pens to the distribution shapes. Each regression model was assessed using root mean squared error (RMSE) on its own training–testing data, a held-out 25% of the pens assigned to its distribution family.

The unit of interest in this study was the pen. The XGB algorithm was selected from a set of machine learning algorithms in preliminary comparisons. Classification performance was evaluated by accuracy, precision, recall and F1-score with Equations (1)–(4).

(1) $$\mathrm{Accuracy}\;(\frac{\mathrm{True Positives}+\mathrm{True Negatives}}{\mathrm{True Positives}+\mathrm{True Negatives}+\mathrm{False Negatives}+\mathrm{False Positives}})$$

(2) $$\mathrm{Precision}\;(\frac{\mathrm{True Positives}}{\mathrm{True Positives}+\mathrm{False Positives}})$$

(3) $$\mathrm{Recall}\;(\frac{\mathrm{True Positives}}{\mathrm{True Positives}+\mathrm{False Negatives}})$$

(4) $$\text{F1-score}\;\;\;\;\;\;2\;\times \;(\frac{\mathrm{Precision}\times \mathrm{Recall}}{\mathrm{Precision}+\mathrm{Recall}})$$

The classification and regression XGB algorithms were next evaluated to examine the effect of additional pen features on performance. The base feature set included: pen size, parity, WIM, and milk yield descriptive statistics. The algorithms were trained again with the addition of milk protein and milk fat descriptive statistics, followed by the further addition of BW and BCS descriptive statistics. For both additional feature sets, performance was evaluated by skill scores and RMSE and compared to the scores of the base for selection of the best feature set. Both the preliminary algorithm selection and feature set comparisons used five-fold cross-validation within the 75% training subset. Classification skill scores and RMSE for the distribution parameters were calculated on the held-out fold in each iteration and averaged across the five folds.

Hyperparameters were the properties of each XGB algorithm that affected the decision process, including the depth of decision trees, learning rate, number of boosting rounds, and proportions of pens and features sampled. The DSM algorithms were tuned using Random Search cross-validation with the “RandomizedSearchCV” function of Scikit-learn [21]. For each, 50 hyperparameter combinations were evaluated by three-fold cross-validation (Table 3). Hyperparameter combinations were ranked by mean squared error (MSE) averaged across the three folds.

We evaluated the DSM using the independent testing dataset [9]. For each pen a distribution shape and its parameters were predicted by the XGB models, resulting in a PDF for the DMI of each pen. We simulated individual cow DMI values from the pen’s PDF, and drew observations equal to the pen size. Summing these values calculated the predicted total pen DMI. This process of randomly drawing observations from the PDF was done 100 times per pen with the mean of 100 predicted pen totals used as the final pen total DMI.

To compare the DSM calculation of pen DMI to a conventional method, the DMI of each pen in the testing data was predicted by the NSM using (5) from [4] and (6) from [22]

*DMI* = [(3.7 + *parity* × 5.7) + 0.305 × *MilkE* + 0.022 × *BW*(*kg*) + (−0.689 + *parity* × −1.87) × *BCS*] × [1 − (0.212 + *parity* × 0.136) × *exp*^(−0.053 × *DIM*)^] (5)

*MilkE concentration* = ((0.0929 × *milk fat* (%)) + (0.0547 × (*milk protein* (%) ÷ 0.93)) + 0.192) (6)

The input parameters of the NSM included milk energy, days in milk (DIM), parity, BW and BCS. Cow BCS was not recorded in the Lean testing dataset, so this value was fixed at 3. All other inputs were set at the mean of each pen, and a DMI value was calculated. The calculated DMI was multiplied by the number of cows in each pen for the pen DMI estimate. Pen DMI predictions were compared to the observed, and model diagnostic evaluation was performed.

Predicted pen DMI was compared to the observed DMI of every pen in the testing dataset using mean squared error of prediction (MSEP) partitioned into error due to mean bias, error due to the slope not equal to 1 and error due to random variation. The coefficient of determination (R^2^), root mean squared error of prediction (RMSEP), RSR (ratio of RMSEP/standard deviation), concordance correlation coefficient (CCC), correlation coefficient (r) and bias correction factor (Cb) were calculated to evaluate model performance [23].

## 3. Results

### 3.1. DSM Development

The DSM used pen features to predict the DMI distribution (Beta/Generalized Normal) with an XGB classification algorithm, and its parameters with separate XGB regression models. Total pen DMI was estimated by summing simulated individual cow intakes within the predicted distribution (the mean of 100 simulations was the final result). Performance of the DSM was evaluated for distribution classification, parameter prediction, and total pen DMI.

#### 3.1.1. Feature Set Selection

The XGB algorithms were compared using three feature sets by five-fold cross-validation (Table 4). Classification accuracy was approximately 0.71 for all three feature sets, and adding milk fat, milk protein, BW and BCS produced little change in classification or distribution-parameter prediction performance. The base feature set of pen size, parity, and descriptive statistics of WIM and milk yield was therefore selected for the final classification and regression models.

#### 3.1.2. Features of the DSM

Feature importance was ranked by gain, the average improvement in the training objective achieved by splits using each feature (Table 5). For the classification algorithm, the parity category of a pen was one of the top three features. This was expected, as primiparous and multiparous cows have systematically different DMI due to growth requirements and lower milk yield and size. Since Beta can represent skewed distributions, this may explain parity category being the most important feature in classification between the two distribution candidates. For the non-linear regression algorithms, parity category was in the top four most important features. The maximum and minimum milk yield were also some of the top features, as they influence the spread of DMI data. Pen size was one of the top three features for the Beta regression algorithm, but the fourth lowest feature of importance for the Generalized Normal regression algorithm. This may indicate that pen size had a large importance in fitting non-normal or skewed distributions such as the Beta.

#### 3.1.3. Hyperparameter Tuned XGB Algorithms

The XGB was evaluated with the training–testing data to assess overfitting, and the testing data to assess model performance on previously unseen data (Table 6). The classification algorithm had an overall accuracy of 0.71 on training–testing data. Accuracy is the total number of correctly classified instances over the total data and is a simple measure of correctness. Good classifiers have values close to 1 for both precision and recall. The classifier only had good performance for the Generalized Normal distribution shape.

**Figure 1:**
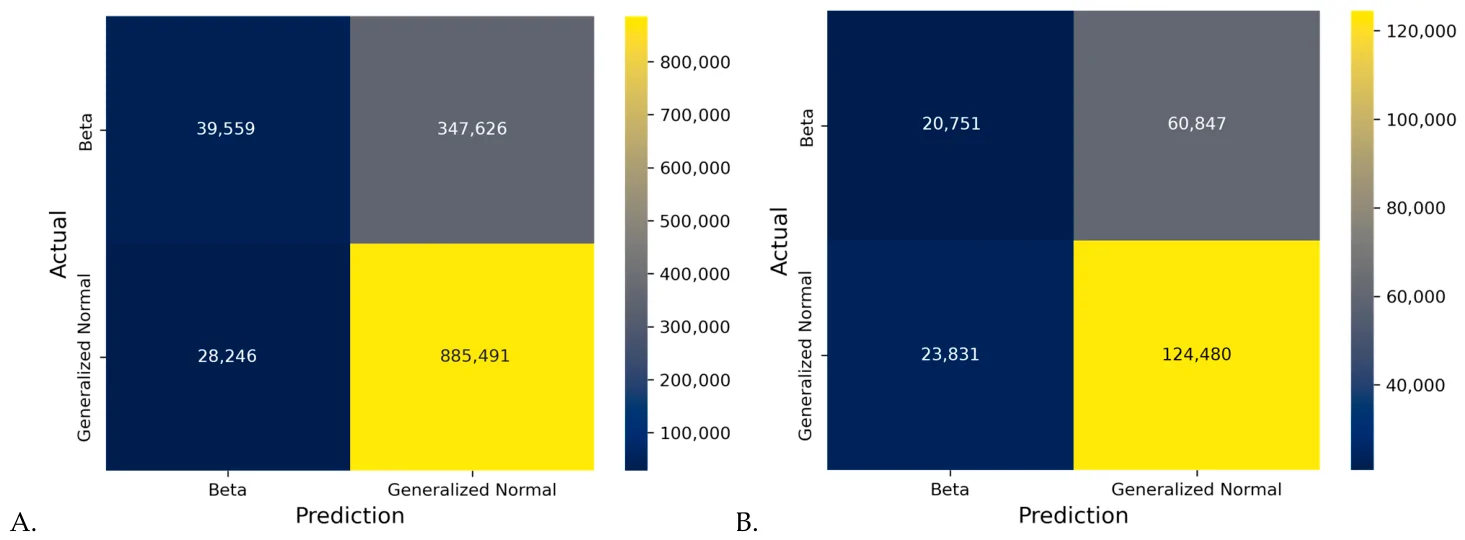
A confusion matrix of the actual and predicted classification of the XGB trained and hyperparameter tuned model for the training–testing data (**A**) and testing data (**B**). True positives and negatives are the quadrants with a matching class for both actual and prediction. False positives and negatives are the quadrants where the class of actual and prediction are mismatched.

Beta was the minority class (30% of the training–testing pens). Most Beta pens were misclassified as Generalized Normal (347,626 false negatives, 39,559 true positives), and most Generalized Normal pens were classified correctly (885,491, with 28,246 misclassified as Beta; Figure 1A). Generalized Normal had high skill scores for classification with an F1 of 0.82 (Table 6), and classification was biased towards this majority class. The RMSE of parameter prediction on the training–testing data was 10.98 for Beta and 0.46 for Generalized Normal (Table 6). The full testing dataset was 229,909 pens, of which 148,311 were labeled Generalized Normal, and 81,598 (35%) were labeled Beta. The overall classification accuracy on the testing data was 0.63 (Table 6). Compared with the training–testing data, Beta recall and F1-score were higher on the testing data, but Beta precision was lower (Figure 1B).

#### 3.1.4. DSM Prediction of Pen DMI with Testing Data

The DMI prediction of pens classified as Beta had a high R^2^ and an RMSEP of 72.5 kg/d (6.9% of mean observed pen DMI), and almost all model error was due to random variation rather than mean bias, or the regression slope not equal to 1 (Table 7). The RSR, CCC, r and Cb all indicated high precision and accuracy. The same was observed for pens classified as Generalized Normal, with a high R^2^, an RMSEP of 45.7 kg/d (4.0% of mean observed pen DMI), and most error due to random variation. The MSEP of the Generalized Normal pens in the model was lower than for the Beta classified pens and had RSR closer to 0, and all diagnostics indicated high performance for both groups. RMSEP was 51.5 kg/d for pens classified into the correct distribution family and 52.7 kg/d for misclassified pens.

#### 3.1.5. NSM Prediction of Pen DMI with Testing Data

**Figure 2:**
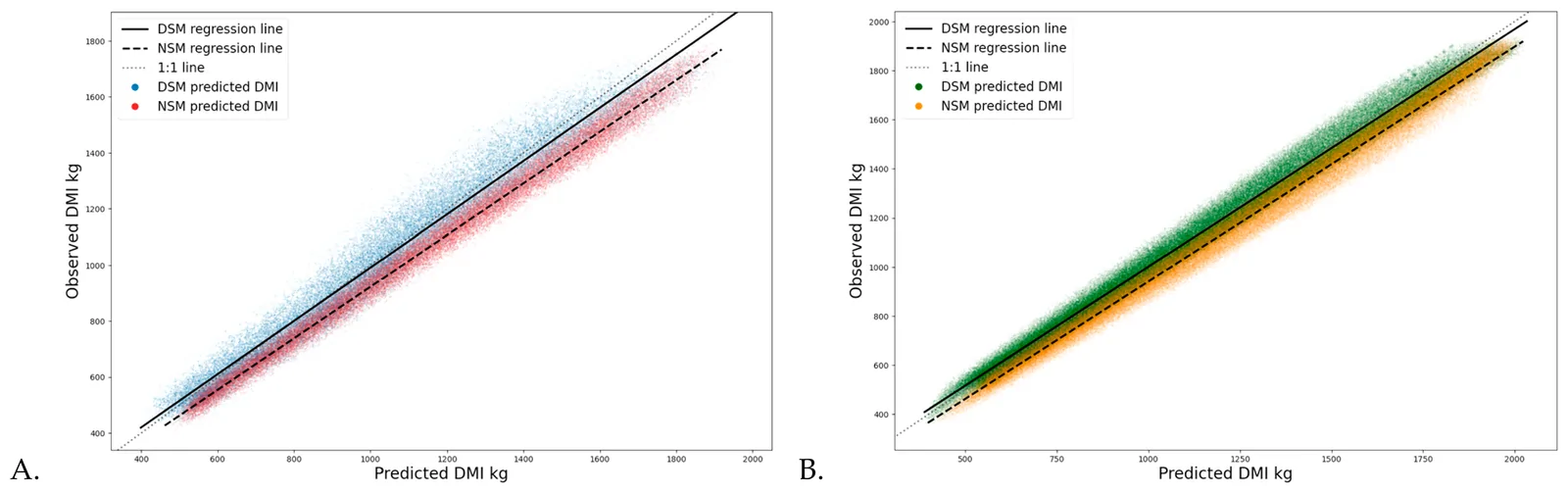
Observed versus Predicted plots of DMI by pen for Beta distribution pens (**A**), or Generalized Normal pens (**B**) for both the DSM and NSM. Solid and dashed lines are least-squares regressions of observed on predicted DMI for each model; the dotted line is the 1:1 line.

For pens classified as Beta, the NSM prediction of pen DMI had an R^2^ of 0.913 and an RMSEP of 98.7 kg/d (9.4% of mean observed pen DMI), and most error was due to mean bias (80.2%) rather than random variation (Table 7, Figure 2). The NSM overpredicted pen DMI by an average of 88 kg/d (1.7 kg/cow/d). For pens classified as Generalized Normal, R^2^ was 0.956 and RMSEP was 80.2 kg/d (7.0% of mean observed pen DMI), and most error was again due to mean bias (73.2%), with an average overprediction of 69 kg/d (1.2 kg/cow/d).

#### 3.1.6. Comparison of DSM and NSM

The NSM had higher prediction error than the DSM for both distribution groups, 1.9-fold higher MSEP for pens classified as Beta (9736 vs. 5249 kg^2^) and 3.1-fold higher MSEP for pens classified as Generalized Normal (6431 vs. 2086 kg^2^) (Table 7). RMSEP was 26.2 and 34.5 kg/d higher for the NSM, respectively. Most NSM error was due to mean bias, reflecting consistent overprediction of pen DMI (Figure 2), whereas most DSM error was due to random variation. The DSM had lower RSR and higher R^2^, CCC and Cb than the NSM in both groups. Correlation coefficients were high for both models (r ≥ 0.978), indicating that NSM predictions followed differences in observed pen DMI but were systematically too high. MSEP values were large in absolute terms because pen DMI increases with pen size.

## 4. Discussion

### 4.1. DSM

#### 4.1.1. Base Feature Set of the DSM

Adding milk fat, milk protein, BW and BCS descriptors to the base feature set changed classification accuracy and parameter RMSE only slightly, although Beta recall and F1-score were modestly higher with more features. Most of the predictive information came from pen size, parity and WIM, which are routine pieces of pen information, and milk yield, whereas BW and BCS are less commonly recorded. Selecting the base feature set accepted a small loss in Beta classification in exchange for a model that can be applied with readily available records and does not rely on imputed BCS values. Feature importance rankings describe how the fitted models used each feature rather than causal relationships, and importance measures based on tree splits can favor features with many possible split points. However, the parity categories, which allow only one split each, ranked among the most important features in all three models.

#### 4.1.2. Distribution Fitting and Pen Exclusions

Parameter-based exclusions removed 56% of pens labeled Beta but only 0.05% of pens labeled Generalized Normal. The excluded Beta fits had extreme parameter values and did not describe a plausible DMI curve. The Beta distribution has four parameters and can take a wide range of shapes, so these values may reflect pens whose DMI distribution had no clear shape, particularly among smaller pens. Each pen was fitted only with the two distributions selected in the preliminary analysis, so excluded pens were not necessarily poorly described by all candidate distributions. The DSM was therefore built and tested only on pens with plausible Beta or Generalized Normal fits, and may be less accurate for pens with other DMI distributions. Removing these pens also changed the training data from roughly equal numbers of Beta and Generalized Normal pens (49% Beta) to 30% Beta, which likely contributed to the low Beta recall of the classifier.

#### 4.1.3. Performance of the Hyperparameter Tuned XGB Algorithms

Classification was the weakest part of the DSM. In the testing data, most Beta pens were classified as Generalized Normal. This was likely related to the overlap between the two candidates. The Beta can represent skewed data but can also describe data similar to a normal distribution, while the Generalized Normal describes symmetric data with flexible kurtosis and data concentrated around the mean. This made the Generalized Normal appropriate for livestock management as our practical goal should be to group animals by similarity in nutrient requirements, which concentrates pen DMI around the mean. Many pens could therefore be described reasonably well by either distribution. Pen management can also introduce skew systematically, for example when first-lactation cows are grouped with multiparous cows, and the Beta can describe these pens. This is consistent with parity being among the most important features for classification and with Beta and Generalized Normal being the most frequent shapes in the preliminary analysis. Neither distribution describes multimodal data, which could occur in pens with both primiparous and multiparous cows because of their different energy requirements. However, DSM predictions of total pen DMI had similar error for correctly and incorrectly classified pens, so misclassification between these overlapping shapes had little effect on the final pen DMI prediction.

On the training–testing data, parameter RMSE was 10.98 for the Beta model and 0.46 for the Generalized Normal model. The Beta parameters ranged from 0.32 to 80 for α, 0.38 to 50 for β, −60 to 22 for location and 6.8 to 161 for scale, and the Generalized Normal parameters ranged from 0.32 to 33 for shape, 12 to 28 for location and 0.033 to 12 for scale. Because RMSE was averaged across each model’s parameters, it was larger for the Beta model, whose location and scale parameters covered much wider ranges. The two RMSE values therefore cannot be compared directly, and the effect of parameter error was assessed using total pen DMI in the testing data.

#### 4.1.4. Comparison of DSM and NSM Prediction of Pen DMI with Testing Data

The NSM overpredicted DMI for pens classified as both Beta and Generalized Normal (Figure 2), while the DSM predicted DMI close to observed, with a smaller overprediction as pen size increased. The NSM predicted DMI for one average cow per pen. Because DMI increases rapidly in the weeks after calving, fresh cows eat much less than an average cow would, so this approach can overestimate the DMI of early-lactation pens, which may explain the consistent NSM overprediction in these testing pens. For pens classified as Generalized Normal, both models overpredicted on average, but the bias of the DSM was much smaller than that of the NSM (4 vs. 69 kg/d). The Generalized Normal can describe DMI more tightly clustered around the mean than a normal distribution, whereas the NSM assigns the same DMI to every cow and cannot describe variation among cows within a pen.

The NSM was applied in a simplified way, using pen-average inputs and a fixed BCS of 3. This is akin to the real-world application where pen averages are commonly used. A more robust approach would be to calculate the DMI for each cow in every pen by this equation and sum those values. This was not possible here because individual cow information was not retained for the 229,909 testing pens, but it would likely improve NSM predictions, particularly for early-lactation pens, although it would be much less feasible in real-world scenarios. The testing pens only included cows up to 15 WIM, the most dynamic phase of lactation, when DMI changes rapidly as cows approach peak milk yield, making it a challenging period in which to predict DMI. DMI is generally more stable later in lactation, and although the DSM classification and parameter predictions were also evaluated on training–testing pens that included later lactation, total pen DMI and the comparison with the NSM were only tested up to 15 WIM. The testing pens were not independent observations. All 229,909 testing pens were generated from the weekly records of 88 multiparous cows in one study [9], and each cow’s records were reused across many pens. The MSEP, RMSEP, R^2^, CCC and the comparison of the DSM and NSM therefore describe performance across combinations of these 88 cows rather than across independent pens, and the DSM should be tested with pens from other herds.

The consistent overprediction by the NSM illustrates the limitation of representing a pen with an average cow. A mean can be calculated for any distribution, but on its own it does not describe how DMI varies among cows in a pen, such as skew or tails. Understanding the distribution of DMI could support calculating the nutrient quantities required by a pen, rather than a uniform supply for each cow. This could improve nutrient utilization, excretion, precision feeding and diet cost, and merits testing in commercial dairy pens. Formulating a ration to the pen’s requirements could provide a more precise solution than using the mean, 75th percentile [24] or applying a correction factor to the ration. These approaches are a crude scaling of quantity, rather than the optimized solution appropriate to the correct requirements. When solving a ration with an optimizer, such as a least cost, or maximizing income-over-feed cost, the solution of ingredients and their quantities will be different at different DMI constraints. If a pen DMI that accounts for the distribution among cows is more accurate, the resulting ration would differ and could reduce the gap between the feed required and the feed provided. If the distribution of DMI is unknown and a pen ration is solved with a single cow DMI, it may not be the optimum ration for the pen.

Both the Beta and Generalized Normal distribution shapes appropriately predicted pen DMI, and both can describe data clustered around the center. The goal of dairy management is to group cows in pens that are similar in reproductive status, production, and nutrient need. In a normal distribution, 95% of the data fall within two standard deviations of the mean, but if DMI is skewed or has heavy tails, a single average cow, as used in the NSM, will not represent the cows in the pen. Starting first with a prediction of the expected shape of DMI distribution based on pen descriptive statistics, then calculating a pen DMI from this shape, the DSM predicted pen DMI with high precision and accuracy in early-lactation testing pens. Explaining the distribution of DMI by pen could improve the precision of diet formulation, as the input constraint of DMI would be closer to the true value. This study also showed that pen DMI could be predicted from information readily available on a commercial dairy, such as parity, WIM and milk yield, without collecting further data on individual cows.

## 5. Conclusions

A machine learning model (DSM) was developed to predict the DMI distribution shape and total DMI of dairy cow pens using only pen size, parity, WIM and milk yield information. The DSM classified the distribution shape and accurately predicted total pen DMI in testing pens. A similar model of DMI prediction, NSM, was also tested and assumed a uniform DMI of cows in a pen. The NSM overpredicted pen DMI, with 1.9 to 3.1 times the MSEP of the DSM, mostly due to mean bias. Predicting the distribution of DMI with the DSM provides a DMI constraint that could be used to formulate a precise ration for a pen. A different optimum ration can be solved for each pen using this predicted distribution rather than scaling an approximated cow DMI value to the number of cows in that pen and assuming uniform DMI by cow.

## Figures and Tables

**Table 1:** Description of the cow records from the separate datasets used to build the database of this study.

Reference	Total ^1^	Primi ^2^	Multi ^3^	Breed	WIM	DMI ^4^
[10] ^5^	55	15	40	Holstein	1–44	Calan gate
[11] ^6^	74	29	45	Holstein	1–36	Calan gate
[12] ^7^	37	0	37	Holstein & Jersey	1–8	Calan gate
[13] ^7^	45	0	45	Holstein	3–18	Calan gate
[14] ^7^	40	0	40	Holstein	1–21	Calan gate
[15] ^7^	31	0	31	Holstein	1–10	Calan gate
[16] ^5^	36	12	24	Holstein	3–43	Calan gate
[17] ^7^	57	57	0	Holstein	1–9	Calan gate
[18] ^5^	308	119	189	Holstein	1–48	Calan gate
[9] ^8^	88	0	88	Holstein	1–15	Calan gate
	771	232	539			

^1^ Cow population per trial before exclusions. ^2^ Primiparous cows in their first lactation. ^3^ Multiparous cows. ^4^ DMI recording method, American Calan, NH, USA. ^5^ Dataset recorded weekly average dry matter intake, milk, milkfat, milk protein and bodyweight. ^6^ Dataset recorded weekly average dry matter intake, milk, milkfat, and milk protein. ^7^ Dataset recorded weekly average dry matter intake, milk, milkfat, milk protein, bodyweight, and body condition score. ^8^ Dataset reserved for testing and was not included in model development.

**Table 2:** Pen level bounds for assortment from individual cow weekly observations.

	Value Range			n of Pens
Pen Type	Earliest Week	Latest Week	Pen Size ^1, 2^	
Fresh	1	3, 4, 5	25–100	10,360
Early	1	6–41	25–250	390,300
Peak	3–16	8–41	50–250	4,092,140
Late	10–20	18–45	50–250	2,698,240
Total				7,191,040

^1^ Increments of 1. ^2^ All pens were assorted with primiparous cows only, multiparous cows only, or both.

**Table 3:** Hyperparameter search ranges and fitted XGB model settings.

Hyperparameter	Search Values	Classifier	Beta Regression	Generalized Normal Regression
Maximum tree depth	3, 5, 6, 10, 15, 20	10	6	10
Learning rate	0.01, 0.1, 0.2, 0.3	0.01	0.01	0.01
Boosting rounds	100, 500, 1000	1000	1000	1000
Proportion of pens sampled	0.5–0.9, step 0.1	0.5	0.8	0.9
Proportion of features sampled per tree	0.4–0.9, step 0.1	0.5	0.7	0.8
Proportion of features sampled per tree level	0.4–0.9, step 0.1	0.7	0.4	0.4

**Table 4:** Performance of the XGB classification and regression algorithms with different feature sets by 5-fold cross-validation.

XGB Algorithms:	Milk Only ^1^	Milk and Solids ^2^	Full Feature Set ^3^
Classification ^4^			
Overall accuracy	0.710	0.712	0.712
Beta			
Precision	0.566	0.567	0.567
Recall	0.110	0.128	0.135
F1-score	0.184	0.209	0.218
Generalized Normal			
Precision	0.719	0.722	0.723
Recall	0.964	0.959	0.957
F1-score	0.824	0.824	0.824
Parameter regression			
Beta RMSE	10.984	10.966	10.960
Generalized Normal RMSE	0.468	0.461	0.456

^1^ Training features included pen size, parity, and descriptive statistics of milk yield and week in milk. ^2^ Training features included pen size, parity, and descriptive statistics of milk yield, week in milk, milk fat, and milk protein. ^3^ Training features included pen size, parity, and descriptive statistics of milk yield, week in milk, milk fat, milk protein, bodyweight, and body condition score (BCS). ^4^ Values are means across five cross-validation folds, rounded to three decimal places. Accuracy includes both classes; precision, recall, and F1-score are reported separately for each class. RMSE = root mean squared error of distribution-parameter predictions; XGB = Extreme Gradient Boosting.

**Table 5:** Feature importance of the three XGB trained and hyperparameter tuned models.

Rank	Classification	Beta	Generalized Normal
1	Parity: Heifers & Cows	Parity: Heifers only	Parity: Heifers only
2	Parity: Heifers only	Parity: Cows only	Parity: Cows only
3	Parity: Cows only	Pen size	Parity: Heifers & Cows
4	Maximum milk yield	Parity: Heifers & Cows	75th percentile milk yield
5	Minimum WIM	Maximum milk yield	Maximum milk yield
6	25th percentile WIM	Milk yield standard deviation	95th percentile milk yield
7	5th percentile WIM	Minimum milk yield	Mean milk yield
8	Maximum WIM	95th percentile milk yield	Median milk yield
9	Pen size	Minimum WIM	Median WIM
10	95th percentile WIM	25th percentile WIM	25th percentile WIM
11	75th percentile WIM	5th percentile WIM	Milk yield standard deviation
12	Median WIM	75th percentile milk yield	Minimum WIM
13	75th percentile milk yield	Skew of milk yield	5th percentile WIM
14	Minimum milk yield	Median WIM	75th percentile WIM
15	Milk yield standard deviation	5th percentile milk yield	25th percentile milk yield
16	WIM standard deviation	Mean WIM	95th percentile WIM
17	Mean milk yield	95th percentile WIM	Mean WIM
18	25th percentile milk yield	Maximum WIM	Maximum WIM
19	Median milk yield	Median milk yield	5th percentile milk yield
20	Kurtosis of milk yield	Mean milk yield	WIM standard deviation
21	Mean WIM	75th percentile WIM	Pen size
22	95th percentile milk yield	25th percentile milk yield	Kurtosis of milk yield
23	5th percentile milk yield	WIM standard deviation	Skew of milk yield
24	Skew of milk yield	Kurtosis of milk yield	Minimum milk yield

**Table 6:** Model performance of the hyperparameter tuned XGB algorithm with both training–testing and testing data ^1^.

Model Performance	Classification				Continuous
	Accuracy ^2^	Precision	Recall	F1-Score	RMSE
Training–testing data					
Beta		0.58	0.10	0.17	10.98
Generalized Normal		0.72	0.97	0.82	0.46
Both classifications	0.71				
Testing data					
Beta		0.47	0.25	0.33	
Generalized Normal		0.67	0.84	0.75	
Both classifications	0.63				

^1^ Training–testing data were the 25% of the training data held back from algorithm training; testing data were generated from the independent dataset in Table 1. ^2^ Accuracy is calculated on both classification levels.

**Table 7:** Model diagnostics of DMI predictions using the DSM or NSM compared to observed DMI from the testing dataset.

Model	MSEP ^1^	RMSEP ^2^	% Bias	% Slope Not = 1	% Random Variation	R^2 (3)^	RSR ^4^	CCC ^5^	r ^6^	Cb ^7^
DSM										
Beta	5249	72.5	3.0	5.4	91.6	0.953	0.217	0.977	0.978	0.999
Generalized Normal	2086	45.7	0.8	7.6	91.6	0.986	0.119	0.993	0.993	1.000
NSM										
Beta	9736	98.7	80.2	8.3	11.5	0.913	0.295	0.961	0.995	0.966
Generalized Normal	6431	80.2	73.2	4.7	22.1	0.956	0.209	0.979	0.995	0.984

^1^ MSEP = mean square error of prediction. ^2^ RMSEP = root mean square error of prediction. ^3^ Coefficient of determination, R^2^. ^4^ RSR = ratio of RMSEP/Standard deviation. ^5^ CCC = concordance correlation coefficient. CCC = r × Cb. ^6^ r = correlation coefficient. ^7^ Cb = bias correction factor. DSM = distribution shape model. NSM = NASEM DMI equation model.

## Data Availability

The raw data supporting the conclusions of this article will be made available by the authors on request.

## Supplementary Materials

The following supporting information can be downloaded at: https://www.mdpi.com/article/10.3390/ani16203184/s1, Table S1: Candidate distributions considered in preliminary fitting of pen dry matter intake.

## Abbreviations

The following abbreviations are used in this manuscript:

BCS	Body condition score
BW	Bodyweight
Cb	Bias correction factor
CCC	Concordance correlation coefficient
DIM	Days in milk
DSM	Distribution shape model
DMI	Dry matter intake
MSE	Mean squared error
MSEP	Mean squared error of prediction
NASEM	National Academies of Sciences, Engineering, and Medicine
NSM	NASEM DMI equation model
PDF	Probability density function
r	Correlation coefficient
RMSE	Root mean squared error
RMSEP	Root mean square error of prediction
RSR	Ratio of RMSEP to standard deviation
R^2^	Coefficient of determination
WIM	Week in Milk
XGB	Extreme Gradient Boosting
