# Supplementary material for: Predicting the Distribution and Dry Matter Intake of Cow Pens with Machine Learning
Source: Animals (Basel). 2026 Oct 10;16(20):3184. doi: 10.3390/ani16203184 (PMC13700728; doi:10.3390/ani16203184)
Supplement: Supplementary file 1 [file animals-16-03184-s001.zip › animals-4539285-supplementary.pdf]

Candidate distributions for pen dry matter intake

Table S1. Candidate distributions considered in preliminary fitting of pen dry matter intake.

No.	Distribution	SciPy identifier
1	Normal (Gaussian) distribution	norm
2	Beta distribution	beta
3	Exponential distribution	expon
4	Gamma distribution	gamma
5	Generalized Extreme Value distribution	genextreme
6	Logistic distribution	logistic
7	Log-normal distribution	lognorm
8	Triangular distribution	triang
9	Uniform distribution	uniform
10	Fatigue life (Birnbaum-Saunders) distribution	fatiguelife
11	Generalized Gamma distribution	gengamma
12	Generalized normal distribution	gennorm
13	Double Weibull distribution	dweibull
14	Double Gamma distribution	dgamma
15	Right-skewed Gumbel distribution (Extreme Value Type I)	gumbel_r
16	Power normal distribution	powernorm
17	Rayleigh distribution	rayleigh
18	Maximum Weibull (inverse Weibull) distribution	weibull_max
19	Minimum Weibull distribution	weibull_min
20	Laplace (double exponential) distribution	laplace
21	Alpha distribution	alpha
22	Generalized Exponential distribution	genexpon
23	Bradford distribution	bradford
24	Beta prime distribution	betaprime
25	Burr (Type XII) distribution	burr
26	Fisk (Log-logistic) distribution	fisk
27	Generalized Pareto distribution	genpareto
28	Hyperbolic Secant distribution	hypsecant
29	Half-normal distribution	halfnorm

No.	Distribution	SciPy identifier
30	Half-logistic distribution	halflogistic
31	Inverse Gaussian distribution	invgauss
32	Inverse Gamma distribution	invgamma
33	Levy distribution	levy
34	Log-Laplace distribution	loglaplace
35	Log-Gamma distribution	loggamma
36	Maxwell distribution	maxwell
37	Mielke's Beta-Kappa distribution	mielke
38	Non-central Chi-squared distribution	ncx2
39	Non-central F distribution	ncf
40	Non-central Student's t distribution	nct
41	Nakagami distribution	nakagami
42	Pareto distribution	pareto
43	Lomax distribution (Pareto Type II)	lomax
44	Power log-normal distribution	powerlognorm
45	Power-law distribution	powerlaw
46	Rice distribution	rice
47	Semicircular distribution	semicircular
48	Trapezoidal distribution	trapezoid
49	Inverse Weibull distribution	invweibull
50	Folded normal distribution	foldnorm
51	Folded Cauchy distribution	foldcauchy
52	Cosine distribution	cosine
53	Exponential power distribution	exponpow
54	Exponential Weibull distribution	exponweib
55	Wald (Inverse Gaussian) distribution	wald
56	Wrapped Cauchy distribution	wrapcauchy
57	Truncated Exponential distribution	truncexpon
58	Truncated Normal distribution	truncnorm
59	Student's t distribution	t
60	R-distribution	rdist
